# The challenges of physicians’ participation in hospital accreditation programs: a qualitative study in Iran

**DOI:** 10.1186/s12913-021-07182-w

**Published:** 2021-10-28

**Authors:** Hosein Ebrahimipour, Elahe Hooshmand, Mehdi Varmaghani, Javad Javan-Noughabi, Seyyed Morteza Mojtabaeian

**Affiliations:** 1 Social Determinants of Health Research Center, Mashhad University of Medical Sciences, Mashhad, Iran; 2 Student Research Committee, Shiraz University of Medical Sciences, Shiraz, Iran

**Keywords:** Physicians’ participation, Accreditation, Qualitative approach

## Abstract

**Background:**

Due to the increasing pressure on hospitals to enhance the quality of services, the participation of physicians in accreditation programs has become more important than ever. The present study was conducted to describe challenges of physicians’ participation in hospital accreditation programs in Iran using a qualitative approach.

**Methods:**

We conducted interviews with 11 managers, 9 physicians and 8 experts in the field of hospital accreditation. Interviewees were selected through purposive snowball sampling. In-depth unstructured and semi-structured interviews were conducted for data collection. The data obtained were analyzed in ATLAS.ti using the conceptual framework method.

**Results:**

The results of this study extracted 3 main themes including: cultural, organizational and behavioral factors. Also, this study found 12 sub-themes and 57 items. Sub-themes in the cultural domain were motivation, patient demand, mutual trust and evaluation system. The organizational domain consisted of seven sub-themes, including high workload, understanding the role of quality management unit, unrealistic accreditation, nature of accreditation, empowerment of physicians in the field of quality, effective communication, resource constraint. Sub-themes in the behavioral dimension were ambiguity in the role and uncertainty about how to participate in accreditation program.

**Conclusion:**

Physicians’ participation in accreditation programs can be increased through culture building and proper training about accreditation activities in the medical community.

## Introduction

People expect to receive comprehensive, safe and high quality health services and hospitals play an important role in this regard [1]. Hospitals have a serious duty and mission to maintain health and care for the life of the community [2]. The special nature of medical services and therefore the lack of experience of the client in assessing these services, which causes even a mistake within the treatment of patients, imposes very serious consequences on them and costs a lot of money [3].

Therefore, the quality of health services provided by hospitals is very important [2]. For this purpose, hospital accreditation has been introduced as a key strategy for providing safe and quality healthcare. Hospital accreditation is “an external assessment of a hospital’s structures, processes, and results by an independent professional accreditation body using pre-established optimum standards” [4]. In Iran, the Ministry of Health and Medical Education developed the standards of accreditation in 2010 and dispatched them to hospitals. The accreditation program has been implemented in 37 hospital wards and 8104 measures since 2012 to familiarize hospitals with the program and provide the chance for them to get properly prepared [5]. However, the standards were rather structural and process-bound. Furthermore, the number of outcome-related standards was awfully limited. 20 to 25 evaluators of the Vice-Chancellors of Treatment in Universities of Medical Sciences were in charge of accrediting hospitals. In other words, approximately 1200 individuals were involved in the evaluation and accreditation of hospitals throughout the country. In 2014, a revision was brought about and second-generation standards were expressed in 36 sections and 2157 measures. In 2016, the new-generation accreditation standards were developed in 8 axes, 248 standards, and 903 measures [5]. The 8 axes of third-generation accreditation are as follows: management and leadership, infection prevention and control, para-clinical services, care and treatment, information management, respect for the rights of service recipients, management of nursing services, and management of medications and equipment [5]. In Iran, the accreditation process comprises the two stages of internal evaluation and external evaluation. Internal evaluation is the first stage of the accreditation model in which the members themselves formulate the indicators and the evaluation questions. Next, they determine the methods of data collection. Then, they pass judgment on themselves once data collection and analysis are finished. Eventually, we contradicted the current situation with a desirable one. Consequently, the strengths and weaknesses were revealed and strategies and suggestions for improving the quality were proposed and implemented. In other words, the current situation’s level of compatibility with the indicators is evaluated. Based on the results, future activities are planned in internal evaluation [6, 7]. External evaluation is the second stage of accreditation in which the homogeneous board of directors visits the hospital from outside the target hospital. Not only does it review the internal evaluation report, but it also prepares the official evaluation report of the accreditation system. The outcome of this report leads to the ranking of the hospital system [7]. Physicians are the center of every health care organization and they are key elements in key decisions regarding the care of patients [8]. Therefore, hospitals’ efforts to involve physicians in improving patient care are essential as physicians face increasing reimbursement costs and time constraints.

Multiple accreditation standards and measures are directly related to the physicians’ medical specialties. Formulating these cases and completing several evaluation forms require the cooperation of physicians; because they are the only ones who possess that certain level of knowledge and proficiency [7, 9]. Physician participation in clinical leadership and organizational strategies to enhance quality, an important prerequisite for providing safe and high-quality care. As a result, many hospitals have accepted physician interaction as a top strategic priority. It is obtained in order to better understand the key factors of engagement with the physician. Physicians should be involved in quality improvement activities to make systems work more securely and reliably. Previous evidence suggests that safety and quality improvement are higher when physicians are engaged and committed to the system [10].

Meanwhile, various research studies in Iran have identified the lack of participation on physicians’ end in the accreditation process as one of the most significant challenges in establishing accreditation standards [7, 9]. Physician training programs focus almost entirely on knowledge and skills for patients treatment and there is almost no training in health service management skills or effective quality improvement [11]. It is reported that many problems of physicians’ non-participation can be solved by properly training them, creating appropriate motivation and facilitating their future participation [12]. The article by Mainz et al. showed that physicians’ resistance to quality assurance and quality improvement procedure and hospital accreditation is common in all countries and public health systems [13].

Therefore, since it is very important to improve the quality of health care services in accordance with accreditation standards and increase the participation of physicians in this matter, this study aimed to assess the challenges physicians’ participation in accreditation programs in hospitals affiliated to Mashhad University of Medical Sciences (MUMS) in Iran.

## Methods

This qualitative study was conducted in hospitals affiliated to MUMS in 2020 in Iran. Mashhad, the center of Khorasan-e-Razavi province, is located in northeast of Iran. This city, with a population of about 3 million, is the second largest city in Iran and attracts more than 25 million pilgrims annually.

11 public training hospitals of MUMS participated in the research. The 11 managers from 11 hospitals along with 9 physicians, and 8 experts who had sufficient knowledge about accreditation programs were recruited via purposive snowball sampling for the study. The inclusion criteria included the following: Having effective experience in the field of accreditation (more than one year), which means work experience in a hospital. Having relevant scientific background and participating in accreditation committees (more than 6 months). Data collection continued until there was no new data and no new themes; therefore, we reached data saturation. A total of 28 interviews were conducted prior to data saturation, which was established by consensus among two investigators. The data were collected through in-depth, semi-structured interviews. The first two interviews were in-depth and unstructured. Subsequent interviews tended toward semi-structured interviews. The author began the data collection with a general question (such as “What are the challenges of physicians’ involvement in hospital accreditation activities?”), incorporating the ideas and perspectives of all participants. He then shifted the discussion from broad concepts to more specific topics. The interviews lasted 40–70 min and were conducted in the workplace of the interviewee to make respondents feel more comfortable. The data obtained in ATLAS.ti version 5 were analyzed by the responsible author using the content analysis method. Content analysis consists of five steps including: familiarization, inducing themes, coding, elaboration, and interpretation and checking [14, 15].

Interviews were orally transcribed and reviewed several times to give a general overview. Identify meaningful units or primary codes, classify codes based on similarities and differences, and set themes as indicators of the organization’s core content. The codes were compared by two researchers and decisions on disputes were made by consensus. The present study has been considered to ensure the validity and accuracy of the data from four criteria (validity or acceptability, reliability or compatibility, transferability, verifiability) proposed by GABA and Lincoln [16] and the researcher has tried to make the findings reflect the real experiences of the participants. In-depth interviews, meetings with experts were held to ensure the correctness of the codes and the researcher’s interpretations of them, and correction of codes that do not reflect the opinions of the respondents. The process of subject encryption, classification and extraction was reviewed by supervisors, consultants and experts to ensure that the classes were consistent with the participants’ statements. In order to increase the transferability of the findings and help others in tracking the researchers’ thoughts and examining the characteristics of the study population, the researcher used a clear, accurate and purposeful explanation of the study process. All participants provided written informed consent form for the various aspects of data collection. The Medical Ethics Committee of MUMS has approved this research (No. IR.MUMS.REC.1398.263).

## Results

Most of the participants in this study were men (78%), with a doctorate degree and higher (67%) with 11 to 20 years of work experience. Socio-demographic characteristics of participants are shown in Table 1.

**Table 1:** Demographic characteristics of participants

Demographic characteristics		Health managers (%)	physicians (%)	experts in the field of hospital accreditation (%)	Total (%)
Gender	Female	1 (9)	2 (22.3)	3 (37.5)	6 (21.4)
	Male	10 (91)	7 (77.7)	5 (62.5)	22 (78.6)
education	MSc	3 (27.2)	0 (0)	6 (75)	9 (32.1)
	PhD and above	8 (72.7)	9 (100)	2 (25)	19 (67.9)
work experience	1–10 years	1 (9)	4 (44.4)	3 (37.5)	8 (28.6)
	11–20 years	4 (36.3)	4 (44.4)	4 (50)	12 (42.8)
	21–30 years	6 (54.5)	1 (11.2)	1 (12.5)	8 (28.6)

We identified 3 themes, 12 sub-themes and 57 items for challenges of physicians’ participation in accreditation activities in hospitals. Three main themes were divided into cultural, organizational and behavioral. Sub-themes included motivation, patient demand, mutual trust and evaluation system, high workload, understanding the role of quality management unit, unrealistic accreditation, nature of accreditation, empowerment of physicians in the field of quality, effective communication, resource constraint, ambiguity in the role and uncertainty about how to participate in accreditation program. The categories of themes, sub-themes and items that obtained in this research are shown in Table 2.

**Table 2:** Challenges of physicians’ participation in accreditation programs in hospitals in eastern Iran based on qualitative method

The main concepts	Sub-concept	Items
Cultural	Motivation	➢ Lack of connection between accreditation and physician performance
		➢ Lack of incentives
		➢ Non-compliance of the evaluation results with the actual performance of the hospital
		]➢ Lack of distinction between positive practices in the field of accreditation
		➢ Lack of perceived rewards for participating in accreditation for physicians
		➢ Lack of perceived hospital ratings based on accreditation score
	Patient Demand	➢ A false impression of the patient’s judgment about services
		➢ Lack of sense of demand in patients
	Mutual trust and evaluation system	➢ Low focus of management team on physicians
		➢ Inadequate monitoring systems to monitor physician involvement
		➢ Lack of confidence in management
		➢ No requirement for physicians to participate in the process of accreditation by managers
		➢ Lack of capable managers in the hospital
Organizational	High workload	➢ The contrast between quality and quantity in public hospitals
		➢ High volume of work in public hospitals
		➢ Public or private hospital
		➢ Improper referral system
		➢ Existence of many patients due to the reputation of the hospital
	Understand the role of the quality management unit	➢ Not understanding the accreditation requirement
		➢ Lack of knowledge about the nature of accreditation
		➢ Lack of understanding of the importance of accreditation by physicians
		➢ Inefficiency of the quality improvement office in attracting the participation of physicians
		➢ Lack of common language between people involved in the accreditation process
	Unreality of accreditation	➢ Negative effect of the evaluator
		➢ Non-compliance of accreditation criteria with the actual performance of the hospital
		➢ Lack of transparency of accreditation metrics
		➢ Lack of accreditation criteria based on different medical specialties
		➢ The unity of the evaluator and the evaluated entity
	Nature of accreditation	➢ Separation of hospital accreditation from educational accreditation
		➢ High volume of documentation in accreditation
		➢ Time-consuming accreditation process
		➢ Lack of attention to the nature of the species team in accreditation
		➢ Early reversal of general accreditation policies
		➢ Non-continuous accreditation
		➢ Paper Game Knowing accreditation
		➢ Mandatory nature of the accreditation process
		➢ Stressful nature of accreditation
		➢ The nature of the validation test
		➢ The non-competitive nature of accreditation
	Empowering physicians in the field of quality	➢ Lack of familiarity of specialized assistants with accreditation
		➢ Lack of training in the process of quality improvement and accreditation in retraining courses
		➢ Lack of training in the process of quality improvement and accreditation during education
		➢ Inadequate skills of physicians to participate in accreditation
		➢ Ineffectiveness of trainings related to the process of quality improvement and accreditation
	Effective communication	➢ Non-compliance of the hospital information system with the needs of physicians
		➢ The lack of a communication channel between physicians and the Office of Quality Improvement
		➢ The lack of a communication channel between physicians and managers of hospitals
	Resource constraints	➢ Equipment limitations
		➢ Limited human resources
		➢ Limitation of physical resources
		➢ Limited financial resources
Behavioral	Ambiguity in the role	➢ Ambiguity in the role of the physician in the accreditation process
		➢ Doctor of several hospitals
		➢ Multi-occupational physician
		➢ Doctor’s lack of commitment to the hospital
		➢ Lack of proper understanding of job duties
		➢ Lack of sense of responsibility for tasks
	Uncertainty about how to participate	➢ Feel violation of the autonomy of doctors
		➢ Ignoring accreditation
		➢ Sense of cost imposition
		➢ Get used to past trends
		➢ Lack of prioritization of accreditation for the physician
		➢ A view based on the separation of accreditation from clinical practice
		➢ Lack of feeling the need for accreditation
		➢ Uncertainty about the continuation of the accreditation program in its current form
		➢ Accreditation is not institutionalized in organizational culture
		➢ Existence of a sense of Nepotism in the accreditation process

## Cultural challenges

### Motivation

One of the challenges in this area is the lack of connection between the fate and income of the physicians and their performance in accreditation programs.

“The governance and leadership of a medical center has failed to approve processes in which physicians are involved in accreditation. It means that the physician should know that their performance should be in accordance with the accreditation criteria. Otherwise, it will not be able to achieve the desired result”. (E13).

Another common obstacle is the lack of a financial incentive mechanism. Many interviewees stated that the lack of communication between merit pay of physicians and participation in accreditation has greatly reduced contributions.

“If you see that the doctor is involved in the accreditation process and the percentage of her/his fee-for-services goes up, the participation will definitely increase. But when he/she works so hard and gets nothing, he/she says why I should cooperate”. (E22).

Differences in evaluation results with actual performance of hospital are one of the barriers to physicians’ cooperation in hospital accreditation.

“In the big hospitals that always and in any situation ranked first-class hospital, the mentality that may have arisen in doctors is that no matter what conditions they work in and this hospital will always be ranked first because the hospital is a government hospital”. (E28).

Another challenge in this area is the uncertainty of the results of positive performance in the field of accreditation.

“Many of our staff say that we remained in first-class or improved from second-class to first-class but you did not give us any encouragement. It is the same for the doctors. They say that I was so involved in accreditation but I don’t see any results”. (E34).

When hospitals do not reward and punish for taking the initiative in accreditation, the motivation of physicians to participate decreases.

“Another thing is that there is no feedback from this and no incentives for doctors. For example, I came to the hospital’s deputy for treatment and helped in accreditation. What feedback does I get from it, financially or spiritually or in a position?” (E26).

### Patient demand

One of the barriers to participating in accreditation is inability of patients to recognize the quality of services provided by the physicians. Interviewees believed that the lack of a sense of demand among patients play a substantial role in physicians’ participation in accreditation.

“If the patient is demanding, the doctor will be forced to participate more”. (E16).

### Mutual trust and evaluation system

The lack of leadership and proper management of physicians, lack of managerial support for them, and inability to work with other medical professionals were other challenges of physicians’ participation in accreditation.

“Distrust can also play an important role. When a group works together in a manner that benefits everyone, the participation rate will increase. But if they know they are bothering and the management team does not give feedback on their activities, participation will not be important to them”. (E8).

Nonetheless, the management team not obliging hospital physicians to comply with quality and accreditation indicators can diminish their participation in quality and accreditation standards.

“... Perhaps feelings of awkwardness and standing on ceremony are influential as well. Individuals or managers are bashful to confront physicians and participate in accreditation and quality improvement programs. Therefore, they are less inclined to instruct them. Perhaps, even if the managers do instruct physicians, they might be faced with negative reactions”. (E2).

Lack of an accepted evaluation and monitoring framework for physicians for quality and accreditation indicators can lead to a decrease in the level of interest in accreditation standards.

“Participation will be more when monitoring is effective, helpful, and educational”. (E5).

## Organizational challenges

### High workload

One of the biggest challenges of physicians’ participation in accreditation and quality improvement processes in a public hospital is the lack of balance between quantity of physicians’ work and quality of physicians’ work.

“Hospitals care more about quantity than quality. As the income of hospitals increases, they like to provide more services. As a result, quality is sacrificed for quantity”. (E15).

The lack of time and heavy workload for physicians mentioned as a major obstacle in their participation in accreditation programs.

“On the other hand, the problem that doctors have is that they do not have time. Finally, in teaching hospitals, faculty members are so involved in treatment education and research issues that they no longer cooperate in these areas” (E12).

### Understand the role of quality management unit

There is a high degree of indifference among physicians about the need of accreditation for hospital. Also, the lack of knowledge about the implementation of accreditation programs is an another obstacle.

“The most important part is that the doctors did not understand the disadvantages of the lack of an accreditation program”. (E28).

“Many doctors are not involved in this process and they do not know what to do ،many doctors are not aware of the accreditation process at all”. (E9).

### Unrealistic accreditation

It has sometimes been observed that physicians are discouraged and interrogated by the evaluator in performing the accreditation work, which reduces their participation. Also, sometimes the lack of transparency about the collection of accreditation data and the requirements for the use of clinical data, and especially the dissemination of its findings, can discourage physicians from accreditation projects.

“One of the problems with accreditation may be that sometimes the standards or criteria or steps of accreditation are not very clear to the owners of the process and those who work in the hospital”. (E1).

Increasing interventions by the Ministry of Health in relation to quality improvement initiatives are often seen as an another obstacle.

“The government hospitals also tell the Ministry of Health that if you give us a bad grade, you are hurting yourself”. E23).

### The nature of accreditation

Sometimes the multiplicity of methods, tools and approaches related to accreditation processes and quality improvement leads to different types of obstacles to the active participation of physicians. Young physicians feel that these accreditation projects are more paperwork and less involved. In some cases, the periodic and seasonal or discontinuous process of accreditation leads to the fatigue of physicians and their less participation in accreditation projects.

“It means that, unfortunately, the staff understood that quality work means making a document That is, only we are documenting” (E5).

“The accreditation itself, when it came and went, was done for two or three days and stopped, until a year later, two years later, when the accreditation was done again.” (E1).

### Empowering physicians in the field of quality

Many young doctors are not ready for participation in accreditation programs because they do not have the necessary training in their university. Also, on-the-job training about accreditation programs is inadequate.

“At the university, the doctor only learns to treat the disease”. (E28).

### Effective communication

One of the obstacles perceived by physicians at the level of the accreditation system is the lack of a reliable information system and some complex databases that allow the comparison of criteria.

“Now we do not have a single information system, we now have about twenty models of hospital software, each with its own set of problems”. (E2).

### Limitation of resources

The lack of sufficient financial, technical and human resources are other challenges of physicians’ participation in the accreditation programs.

“Nurses, medical equipment and facilities, the hospital environment, the ratio of patients to doctors, all of these are effective for physicians’ participation in the accreditation programs”. (E16).

## Behavioral challenges

### Ambiguity in the role

Despite efforts to improve the hospital accreditation, some physicians still have a vague view of the concept of hospital accreditation.

“Many doctors do not know about their role in accreditation programs”. (E12).

### Uncertainty about how to participate

Physician centeredness is one of the barriers that may lead to the resistance of physicians to participate in accreditation. Many doctors also say that the accreditation programs are new to us and very difficult to do.

“They do not have a critical view and do not accept teamwork”. (E28).

“These programs will not change anything here in the future”. (E8).

Physicians’ involvement in accreditation programs can be challenging. In fact, this culture of accreditation is less common among physicians.

“It is cultured in the nursing system, but not in the medical system. It may be necessary to involve the head manager of medical departments in accreditation work” [12].

## Discussion

The purpose of this study was to identify the challenges of physicians’ participation in accreditation programs in hospitals affiliated to MUMS in Iran. Implementing accreditation requires many changes in the behavior, culture and organizational structure of the hospital to improve the performance of physicians in performing hospital accreditation activities [17]. In this study, the challenges of physicians in accreditation programs are classified into three main dimensions: cultural, organizational and behavioral.

It is reported that there is a lack of culture for participating in teamwork such as accreditation. Based on previous research, physicians feel that accreditation is a formality and are unwilling to participate in the accreditation program [18]. It is suggested that by appropriate modeling of successful countries in the field of hospital accreditation around the world that have outsourced accreditation to the private sector, this is possible to minimize some kind of conflict of interest.

It is reported that the relationship between hospital grade and accreditation score is not tangible. Moreover, it was stressed that it has caused a reduction in motivation and indifference among physicians and treatment staff [19, 20]. One of the solutions is to link the licenses of doctors of a hospital to the rank of that hospital. This helps make the doctors be careful about the rank of the hospital. Another possible solution is to create more coordination between the evaluations by the ministry. One of the other challenges in this regard is that accreditation has no connection with the performances of the physicians, which was also emphasized by Becker and Richard in their research [17]. According to this study, even in countries with a long tradition and successful quality improvement (e.g., the UK), general practitioners (GP) may not have an incentive to introduce ongoing quality improvement initiatives, like accreditation or active search for new opportunities in this field [17]. The future and salary of the physicians must be commensurate with their performance in accreditation.

One of the most notable results of the previous studies was the extent to which the physicians make a difference between their excellent performance in accreditation and the advantages and disadvantages. In the aforementioned research, it was found that the quality improvement activities had a relationship with the discontent of physicians and loss of independence, additional responsibilities, modified reimbursement requirements, and increased expectations of patients, payers, insurers, and regulators [21, 22]. It is recommended to give a special point to it by linking the performance of hospital wards with quality improvement and accreditation activities. Hence, if the department works in accordance with the accreditation rules and regulations, and also the physicians try to enhance the quality of their services, they should receive higher scores than their colleagues. For instance, in the surgery department, those who provide services with higher quality than others should be scored higher [21, 22].

One of the issues, which was also emphasized in a study by Baker, is that the management team does not pay enough attention to the physicians [17]. Based on the results of the above-mentioned research, some of the obstacles of accreditation processes include the lack of leadership of general practitioners and also the lack of managerial support and ability to work with other specialists. Considering that physicians play a very significant role in accreditation, the management team should use more serious levers to involve physicians in accreditation [17]. As mentioned in another study performed by Gleeson, another important issue in this regard is the distrust of management [23]. According to the aforementioned research, the suspicions of physicians about the motivations and covert plans of the managers have prevented them from making the necessary efforts to participate in the accreditation program [23]. Therefore, action should be taken in the hospital to resolve the conflict of interest regarding accreditation and develop a close relationship between the hospital accreditation management team and the physicians. In another study, it is mentioned that young physicians may not be able to access technical advice and support for clinical audits or quality improvement projects if they need help [24]. Considering the significance of the involvement of physicians in the accreditation, it is suggested to add the staff to the quality improvement unit of the hospital to focus more on the enhancement of services provided by the physicians. Another factor mentioned by the participants was unawareness of the nature of accreditation. Another study argued that some of the respondents (physicians) were totally unaware of the accreditation activities [25]. In the aforementioned research, one of the respondents declared that “I perform my clinical activities as I was informed later that our hospital had been evaluated and I was unaware” [25]. It is possible to increase participation by informing the lesson and engaging with the interests and needs of physicians. Other expressed challenges include the lack of transparency and tangibility of the measures. Patow et al. in their research referred to the lack of transparency in the collection of confidential patient data and the conditions of the use of clinical data in research, especially the dissemination of accreditation findings. It can prevent physicians from seeking accreditation projects [26]. Sometimes physicians do not receive information about the accreditation and other quality improvement activities or the necessary feedback. One of the other issues in this area is the recognition of accreditation by physicians. Boyle et al. found that some physicians believe that accreditation requires extra paperwork and takes our time as we should sit and write [27]. One of the most important issues in this area is the obligatory and stressful nature of the accreditation process. Results of the previous studies suggest that physicians avoid involvement or consideration of quality improvement activities due to the fear of being bullied and coerced by evaluators and management teams in accreditation as well as the anxiety and stress of the process. Sometimes negative attitudes of physicians toward accreditation may cause them to view accreditation as a test of their clinical competence and poor performance [28, 29].

Another issue is the lack of education of physicians about quality improvement and accreditation during their studies. In other studies, it is found that in some cases, a gap in knowledge during studies about the relationship between clinical activities and accreditation may cause physicians to perform less than their academic competence and potential for accreditation [30, 31]. As expressed in a study performed by Solberg et al., one of the other significant issues in this regard is the lack of education on the quality improvement and accreditation processes in retraining courses. Based on the results of the aforementioned research, physicians considered on-the-job training about accreditation to be insufficient [32]. It should be noted that the education of physicians for new skills, such as accreditation for measurement, plan development, and quality improvement, needs significant investment; however, most physicians do not have the sufficient capacity for it. Therefore, the training should be assigned to a series of non-governmental organizations; moreover, the University Deputy should supervise them and assess the efficiency of the courses regularly.

One of the other problems is the lack of institutionalization of accreditation in culture. In their research, Hoffimann et al. found that cultural issues can be problematic (e.g., a “culture of blame”) [33]. When cultures prevent people from reporting concerns about patient accreditation and quality improvement, it is difficult to involve physicians in quality improvement processes [33]. To encourage the physicians to participate in these processes, it is suggested to educate physicians about the importance of accreditation for the patients and the physicians themselves. Moreover, the older medical staff of the hospital are recommended to try to improve culture and provide role modeling.

It should be mentioned that the present research has some limitations. The interviewees were selected based on the inclusion criteria using the snowball sampling method; hence, some experts may have been missed. Moreover, it must be noted that the results of this study might have differed if the participants or researchers had been different. Furthermore, this research merely focused on the problems regarding the participation of physicians in accreditation programs, and future studies are recommended to focus on the strengths of the program as well.

## Conclusion

The findings of this study showed that there are three main challenges (cultural, organizational and behavioral) regarding physicians’ participation in accreditation programs. Based on the findings of the present study, policymakers ought to address such challenges regarding successfully engaging physicians in accreditation programs. Hence, it is suggested that cultural barriers be dealt with by establishing motivation and providing managerial support. Then, by creating a balance in the workload of physicians and providing training on the accreditation structure of the hospital and quality improvement units, organizational barriers will be eliminated. Furthermore, by offering education regarding the position and role of physicians in accreditation and also reinforcing the team spirit, behavioral barriers will be considerably reduced.

## Data Availability

The datasets used and/or analyzed during the current study are available from the corresponding author on reasonable request.

## Abbreviations

MUMS: Mashhad University of Medical Sciences
GP: General Practitioners
